# Effects of short‐term hand immobilization on motor functions across domains in adults

**DOI:** 10.14814/phy2.71068

**Published:** 2026-08-20

**Authors:** Lasse Jespersen, August Lomholt Kvistad, Jonas Rud Bjørndal, Jesper Lundbye‐Jensen

**Affiliations:** ^1^ Movement & Neuroscience, Department of Nutrition, Exercise and Sports (NEXS) University of Copenhagen Copenhagen Denmark

**Keywords:** fatigability, fine motor control, force steadiness, immobilization, maximal strength, motor functions, rate of force development

## Abstract

Short‐term limb immobilization is known to impair muscle strength; however, its effects across distinct domains of motor function remain incompletely characterized. In this study, we assessed the effects of 72‐h of hand immobilization on motor functions in healthy adults. Twenty‐eight participants were randomized to an immobilization or control group and completed a motor test battery before and after the intervention. The battery assessed maximal and explosive strength, quantified as maximal voluntary contraction (MVC) force and rate of force development (RFD), fatigability during sustained maximal contractions, force steadiness during submaximal contractions, and manual dexterity. Immobilization induced domain‐specific impairments in motor performance. MVC force and RFD declined significantly following immobilization, indicating susceptibility of maximal and explosive strength to short‐term disuse. In contrast, no significant changes were observed in the selected parameters of fatigability during sustained maximal contractions, including the fatigue time constant and plateau force. Force steadiness showed a selective increase in relative force variability, whereas absolute force variability remained unchanged. Manual dexterity was not reduced from baseline, but immobilization attenuated the test–retest performance improvements observed in the control group and contralateral non‐immobilized hand. These findings demonstrate that hand immobilization induces task‐ and domain‐specific alterations in motor performance rather than a uniform decline, preferentially affecting motor domains that require high force production.

## 1 INTRODUCTION

Limb immobilization is often necessary following injury, but it also provides a robust experimental model for probing adaptive changes in the human motor system. Even brief periods of immobilization induce rapid declines in maximal strength (Campbell et al., 2019), reflecting a cascade of alterations across both the central nervous (Clark, Manini, et al., 2006) and musculoskeletal systems (Clark, Fernhall, & Ploutz‐Snyder, 2006). While the effects of immobilization on maximal and explosive strength are well‐established (Ruggiero & Gruber, 2024), less is known about how immobilization affects other domains of motor control. Current evidence indicates that immobilization induces disproportionate and domain‐specific adaptations, including greater reductions in muscle strength relative to muscle size (Marusic et al., 2021) and faster declines in explosive strength compared to maximal strength (Ruggiero & Gruber, 2024). Given that activity restrictions, such as limb immobilization, are widely used in clinical and rehabilitative contexts despite their adverse consequences, understanding how immobilization affects multiple motor functions can help inform the development of targeted recovery strategies.

Immobilization consistently reduces maximal voluntary contraction (MVC) force (Clark, Issac, et al., 2008; Jespersen et al., 2025; Lundbye‐Jensen & Nielsen, 2008; Valli et al., 2024). However, the ability to generate force rapidly, commonly quantified as the rate of force development (RFD) (Maffiuletti et al., 2016), declines faster during the early phase of immobilization (De Boer et al., 2007; Monti et al., 2021; Sarto et al., 2022), indicating that explosive strength is particularly vulnerable to short‐term disuse (Ruggiero & Gruber, 2024). In addition to strength impairments, immobilization may alter the ability to sustain force over time. Fatigue during sustained maximal efforts reflects a progressive reduction in the neuromuscular system's capacity to generate force (Barry & Enoka, 2007; Gandevia, 2001) and therefore represents a distinct domain of force control. Several studies have examined the effects of immobilization on performance during fatiguing contractions, but the findings have been mixed, potentially due to methodological differences in contraction type (sustained versus intermittent), intensity (submaximal versus maximal), and outcome measures (Boyas & Guével, 2011; Gandevia, 2001). This heterogeneity complicates direct comparisons across studies and leaves the effects of immobilization on fatigability unclear (Clark, Hoffman, & Russ, 2008; Fuglevand et al., 1995; Shaffer et al., 2000).

Immobilization may also affect the finer aspects of motor control, including force steadiness and manual dexterity. Force steadiness refers to the variability observed when attempting to produce a constant force during submaximal isometric contractions (Enoka & Farina, 2021). Although less extensively investigated, some evidence suggests that immobilization increases force fluctuations and contraction variability during submaximal contractions (Inns et al., 2022; Lundbye‐Jensen & Nielsen, 2008; Valli et al., 2024; Yoshitake et al., 2007). Beyond isometric force control, several studies have indicated that immobilization can also impair motor coordination and manual control. For example, upper‐limb immobilization has been shown to reduce kinematic accuracy and disrupt trajectory formation during reaching movements (Huber et al., 2006; Moisello et al., 2008), as well as impair manual dexterity (Opie et al., 2016; Weibull et al., 2011). Together, these findings suggest that the effects of immobilization are not limited to tasks requiring maximal force production but also extend to submaximal force regulation and the coordination of motor behavior.

In summary, the current knowledge of how immobilization affects different motor domains is largely derived from separate studies, each examining distinct aspects of motor performance with varying aims, methodologies, and participant characteristics. Accordingly, the present study aimed to provide a comprehensive behavioral assessment of how short‐term limb immobilization influences motor function across different domains of force and motor control. The participants were tested before and after 72 h of hand immobilization. Focusing on the distal upper extremity allows examination of refined human‐specific actions that require precise finger movements and object manipulation. The hand provides a suitable model for assessing a broad range of motor functions that extend beyond maximal force production. To this end, we employed a motor test battery including measures of maximal, explosive, and fatiguing force production, as well as assessments of force steadiness and manual dexterity. Finally, because repeated motor testing may induce retest effects, a non‐immobilized control group was included to allow changes in motor performance to be interpreted relative to potential practice‐related effects.

## 2 METHODS

### 2.1 Participants

The study included twenty‐eight participants who were assigned to either an immobilization group (IMMO, *n* = 14; mean age ± SD: 26.1 ± 4.3 years; 5 men, 9 women) or a control group (CON, *n* = 14; mean age 24.4 ± 2.2 years; 6 men, 8 women). The sample size (*n* = 28; 14 per group) was determined based on previous studies investigating the functional and neuromuscular consequences of limb immobilization, in which similar sample sizes have been sufficient to detect changes in motor function (Campbell et al., 2019; Marusic et al., 2021; Ruggiero & Gruber, 2024). The present study draws on data from the same participants as a recently published investigation examining neurophysiological and muscular adaptations to short‐term hand immobilization (Jespersen et al., 2025). The data were collected during the same experimental sessions. However, the present study addresses distinct research questions and reports different outcome measures, with only MVC data having been reported previously.

The participants were informed orally and in writing about the experimental procedures and provided written informed consent prior to participation. The study protocol was approved by the Ethics Committee of the Greater Copenhagen Area (approval number: H‐23069733) and was conducted in accordance with the Declaration of Helsinki. The participants were unfamiliar with the test battery before enrollment. All measurements were performed on the dominant hand, which was determined using the Edinburgh Handedness Inventory (Oldfield, 1971).

### 2.2 Experimental approach

**FIGURE 1:**
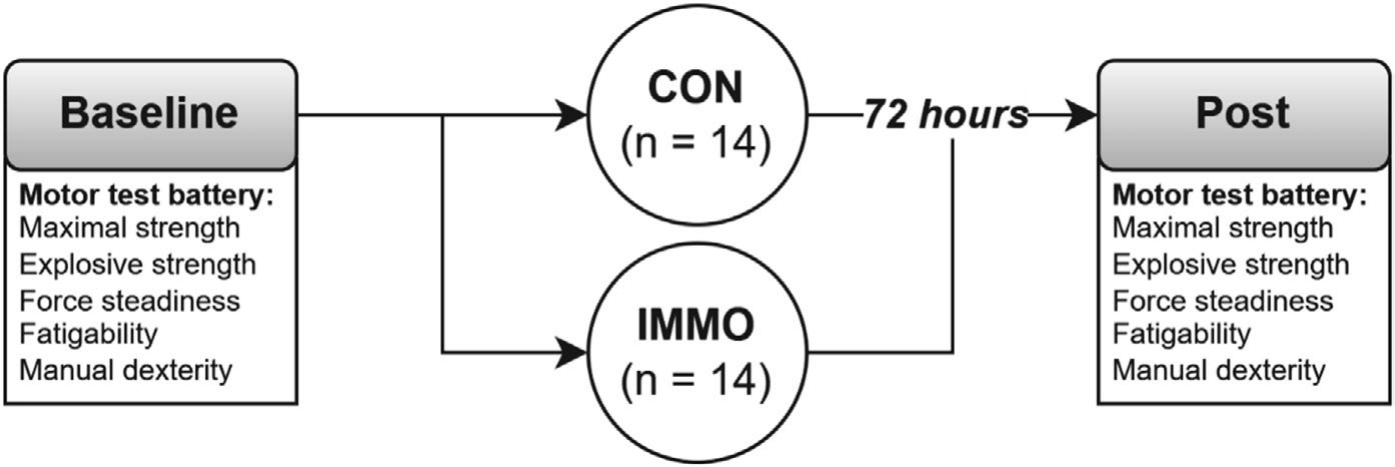
Experimental design and test battery. Schematic overview of the experimental design. Participants were allocated to either a control group (CON, *n* = 14) or an immobilization group (IMMO, *n* = 14). All participants completed the same motor test battery at baseline and again, after 72 h. The test battery included assessments of maximal strength, explosive strength, force steadiness, fatigability, and manual dexterity. Participants in the IMMO group underwent 72 h of dominant‐hand immobilization between testing sessions, whereas the CON group did not undergo immobilization.

The experimental approach and immobilization protocol have been described and illustrated in detail previously (Jespersen et al., 2025). In brief, participants were allocated to either the IMMO group, which underwent approximately 72 h of dominant‐hand immobilization, or the CON group, which participated in the experimental sessions without immobilization. Allocation was based on a predefined randomization schedule generated prior to recruitment. Participants were assigned identification numbers sequentially during enrollment, and group allocation was determined accordingly. Immobilization was achieved using a prefabricated splint secured with a bandage, restricting the movement of the wrist, hand, and fingers. The splint was applied immediately after the baseline session and removed prior to posttesting. A motor test battery assessing performance across multiple domains of motor functions involving assessments of maximal strength, explosive strength, force steadiness, fatigability, and manual dexterity was administered before and immediately after the 72‐h immobilization period or an equivalent control interval (Figure 1).

During the experimental sessions, the participants were seated in an armchair with their tested hand positioned in a custom‐built apparatus. The thumb and index finger were placed around a calibrated load cell, as described by Jespersen et al. (2025). All tasks involving isometric force control were performed using this setup, whereas manual control was assessed using the Purdue Pegboard Test.

The test battery was administered in a fixed order. Maximal and explosive strength were assessed concurrently. Submaximal force steadiness was then evaluated, with the target force defined as a percentage of the peak force obtained during the preceding maximal strength test. Measures of fatigability were obtained by examining the force output during sustained maximal contraction. Finally, the participants were seated at a table adjacent to the testing area, where manual dexterity was assessed using the Purdue Pegboard Test.

### 2.3 Maximal and explosive strength

To assess maximal and explosive strength, the participants performed ten maximal pinch grip contractions, each lasting 2 s, with 1 min of rest between trials. Although it is generally recommended to assess RFD using separate contractions from MVC trials (Maffiuletti et al., 2016), the present study derived RFD from the same contractions used to assess MVC. This was a deliberate trade‐off because separate MVC or RFD trials could have influenced subsequent performance due to fatigue effects. In addition, this approach reduced the total number of maximal contractions required in the test battery, thereby limiting the potential accumulation of fatigue across the broader experimental protocol. Pilot testing conducted prior to data collection confirmed that participants reliably reached maximal force well within the contraction window, consistent with previous findings that maximal force is typically achieved within the first 500 ms of contraction (Rodríguez‐Rosell et al., 2018). Moreover, to maximize the validity of the RFD measurements obtained using this approach, participants were instructed and verbally encouraged to perform each contraction as fast and forcefully as possible (Maffiuletti et al., 2016). Maximal and explosive strength were quantified as maximal contraction force and rate of force development over the initial 0–50 ms and 0–200 ms from contraction onset. Force onset was identified using the repolarization mode in Signal software, with onset defined as the point at which force deviated from baseline by more than 0.2% of the peak force attained during the contraction. A low relative threshold was selected to identify the earliest measurable rise in force while providing an objective and consistent criterion for automated onset detection across all contractions. The 0–50 ms interval was included to characterize the earliest phase of rapid force production, whereas the 0–200 ms interval was included to assess rapid force production over a broader contraction period. In addition, RFD values for both time intervals were normalized to MVC to determine whether changes in rapid force production were independent of changes in maximal force capacity. Visual feedback of the force signal was provided on a monitor placed in front of the participant. Two familiarization trials were performed during the first testing session to ensure that the task instructions were clearly understood.

### 2.4 Force steadiness

Force steadiness was assessed during a submaximal isometric contraction performed at 10% of the MVC. A horizontal target line corresponding to 10% of the highest peak force recorded during the preceding MVC trials was displayed on a monitor in front of the participant. The participants were instructed to match the target force as precisely as possible while minimizing force fluctuations. Each trial began with a 10 s familiarization period, during which the participants gradually reached the target force. The force trace then progressed horizontally from left to right at a constant speed for a 20 s recording interval. Upon reaching the right edge of the screen, the trace was automatically reset to the left, and the procedure was repeated. The task consisted of ten consecutive trials, resulting in a total of 200 s of force recording. Visual feedback of both the force trace and target force was provided continuously throughout the task. Force steadiness was quantified using the standard deviation (SD) of the force signal or the coefficient of variation (CoV%), in which SD is normalized to the mean force, providing measures of absolute and relative force steadiness, respectively (Enoka & Farina, 2021; Pethick et al., 2022). These measures were calculated for each 20 s interval and subsequently averaged across trials.

### 2.5 Fatigability

Fatigability was assessed during a sustained MVC for 200 s. The participants were instructed to apply maximal force to the load cell and maintain this effort for the entire duration of the task. Visual feedback of force output and standardized verbal encouragement were provided throughout the contraction to promote maximal effort. Force data were averaged in consecutive 10 s intervals across the 200 s of contraction. To minimize the influence of onset‐related artifacts, the initial ramp‐up phase was excluded from the analysis. Force was normalized to the mean force of the first complete 10 s interval following contraction onset, which was defined as 100%. Fatigue‐related changes in force were characterized using a one‐phase exponential decay model fitted to normalized force‐time data for each participant. The one‐phase exponential model was selected based on a model comparison performed on baseline data pooled across groups. Individual estimates of the fatigue time constant (*τ*) and plateau force were extracted and used for the statistical analysis. In a small number of cases, the exponential decay parameters could not be reliably estimated. Therefore, *τ* and plateau values were included in the analysis only when the parameter estimates converged with finite 95% confidence intervals. When this criterion was not met, the affected parameter estimates were excluded from the fatigability analyses (CON: *n* = 2; IMMO: *n* = 1).

### 2.6 Manual dexterity

Manual dexterity was assessed using the Purdue Pegboard Test. The participants were instructed to insert as many pins as possible into the designated row within 30 s using their dominant hand. The task was then repeated using the non‐dominant hand of the participant. The number of pins successfully placed during each trial was recorded, and the performance of the dominant and non‐dominant hands was analyzed separately.

### 2.7 Data analysis and statistics

The force signals were amplified (×10), low‐pass filtered at 10 Hz, and sampled at 1000 Hz using a 1401 CED data acquisition system (Cambridge Electronic Design, Cambridge, UK) and analyzed offline using Signal software (version 6.05). Statistical analyses were performed using GraphPad Prism (version 10.2.3).

The present study was designed as an exploratory assessment of the effects of short‐term immobilization on multiple domains of motor function. Accordingly, several outcome measures representing different aspects of motor performance were analyzed separately. For all outcome measures, a 2‐way repeated‐measures ANOVA was conducted with Group (immobilization [IMMO] and control [CON]) as a between‐subject factor and Time (Pre and Post) as a within‐subject factor. When significant main effects or interactions were observed, post‐hoc pairwise comparisons were performed using Fisher's least significant difference procedure, with statistical significance set at *p* < 0.05. Assumptions of normality and homogeneity of variance were assessed visually prior to the analysis.

Data are presented as model estimates with 95% confidence intervals in the Results section, with effect sizes (Cohen's *d*) reported for significant Group × Time interactions based on differences in pre‐ to postintervention changes between the immobilization and control groups. Figures display data using boxplots showing the median, interquartile range, minimum‐to‐maximum values, and individual data points.

## 3 RESULTS

### 3.1 Effects of short‐term immobilization on maximal and explosive strength

**FIGURE 2:**
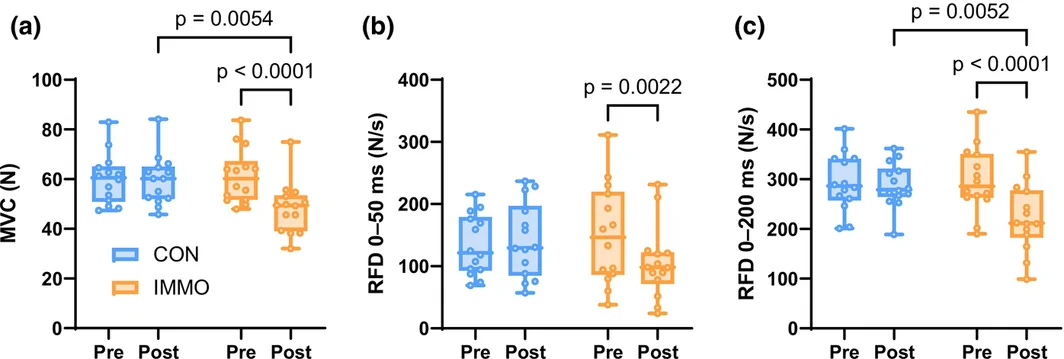
Effects of short‐term hand immobilization on maximal voluntary contraction (MVC) force and rate of force development (RFD). Changes in MVC force (a), RFD over the 0–50 ms interval (b), and RFD over the 0–200 ms interval (c) are shown for the immobilization (orange) and control (blue) groups before (Pre) and after (Post) the intervention. Data are presented as median with interquartile range (boxes) and minimum‐to‐maximum values (whiskers). Individual data points represent individual participants. Exact *p*‐values are shown for statistically significant within‐ and between‐group comparisons.

Analysis of MVC force revealed a significant main effect of Time (*F*(1, 26) = 55.56, *p* < 0.0001), no main effect of Group (*F*(1, 26) = 1.77, *p* = 0.1954), and a significant Group × Time interaction (*F*(1, 26) = 49.60, *p* < 0.0001; Cohen's *d* = 2.66). MVC did not differ between groups at baseline (*p* = 0.7552) and remained unchanged in the control group across Time (*p* = 0.7736). In contrast, immobilization resulted in a significant reduction in maximal strength (mean change: −12.92 N, 95% CI [−15.51, −10.33], *p* < 0.0001; Figure 2a). At post‐test, MVC was significantly lower in the immobilization group compared to the control group (mean difference: −11.33 N, 95% CI [−19.16, −3.49], *p* = 0.0054).

For explosive strength, RFD over the 0–50 ms interval showed a significant Group × Time interaction (*F*(1, 26) = 7.98, *p* = 0.009), with no main effect of Time (*F*(1, 26) = 3.96, *p* = 0.0571) or Group (*F*(1, 26) = 0.20, *p* = 0.657). Baseline 0–50 ms RFD did not differ between groups (*p* = 0.4821), and no change was observed in the control group across time (*p* = 0.5604). In contrast, the immobilization group exhibited a significant reduction in 0–50 ms RFD (mean change: −45.38 N/s, 95% CI [−17.99, −72.77], *p* = 0.0022). No significant between‐group difference was observed at the post‐test (*p* = 0.1314).

Similarly, RFD over the 0–200 ms interval showed a significant main effect of Time (*F*(1, 26) = 49.32, p < 0.0001), no main effect of Group (*F*(1, 26) = 1.93, *p* = 0.18), and a significant Group × Time interaction (*F*(1, 26) = 38.79, *p* < 0.0001; Cohen's *d* = 2.35). Baseline 0–200 ms RFD values did not differ between Groups (*p* = 0.8168), and no changes were observed in the control group across Time (*p* = 0.5788). Immobilization led to a marked reduction in 0–200 ms RFD (mean change: −75.91 N/s, 95% CI [−59.26, −92.57], *p* < 0.0001; Figure 2c). At post‐test, 0–200 ms RFD was significantly lower in the immobilization group compared to the control group (mean difference: −66.08 N/s, 95% CI [−111.50, −20.63], *p* = 0.0052).

To determine whether the reductions in absolute RFD were independent of changes in maximal force, RFD values were additionally normalized to MVC. Following normalization, no significant Group × Time interaction was observed for either the 0–50 ms interval (*F*(1, 26) = 2.30, *p* = 0.1415) or the 0–200 ms interval (*F*(1, 26) = 1.68, *p* = 0.2058).

### 3.2 Effects of short‐term immobilization on fatigability

**FIGURE 3:**
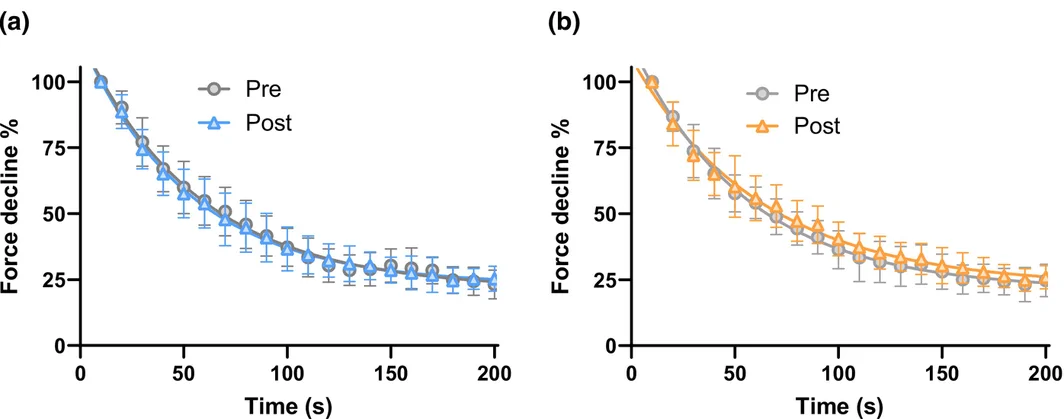
Fatigability during sustained maximal voluntary contraction. Normalized force‐time profiles during the 200 s sustained maximal voluntary contraction are shown for the control (a) and immobilization (b) groups before (Pre) and after (Post) the intervention period. Force values were expressed as a percentage of the mean force during the first complete 10 s interval after contraction onset. Solid lines represent one‐phase exponential fits to the normalized force‐time data. Error bars represent standard error of the mean.

The normalized force‐time profiles during the 200 s sustained MVC with associated exponential fits are shown in Figure 3. Fatigability was quantified using the fatigue time constant (*τ*), derived from one‐phase exponential fits to the normalized force‐time data. The analysis revealed no significant main effects of Time (*F*(1, 23) = 0.0004, *p* = 0.98) or Group (*F*(1, 23) = 0.25, *p* = 0.62) and no Group × Time interaction (*F*(1, 23) = 0.75, *p* = 0.40). Plateau force, expressed as a percentage of the initial force, similarly showed no significant main effect of Time (*F*(1, 23) = 1.03, *p* = 0.32), no main effect of Group (*F*(1, 23) = 0.007, *p* = 0.11), and no Group × Time interaction (*F*(1, 23) = 0.02, *p* = 0.88).

### 3.3 Effects of short‐term immobilization on force steadiness

For absolute force steadiness, quantified as the SD from the force signal, a significant main effect of Time was observed (*F*(1, 26) = 5.06, *p* = 0.033). No main effect of Group (*F*(1, 26) = 0.005, *p* = 0.95) and no Group × Time interaction was detected (*F*(1, 26) = 0.40, *p* = 0.53). Post‐hoc within‐group comparisons were not statistically significant.

**FIGURE 4:**
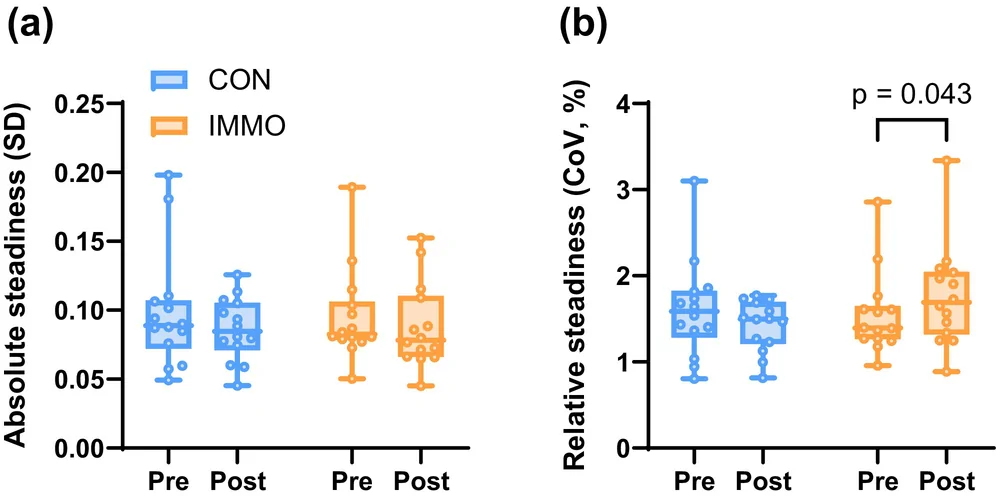
Effects of short‐term hand immobilization on force steadiness. Changes in absolute force steadiness expressed as the standard deviation of force (SD; a), and relative force steadiness expressed as the coefficient of variation (CoV%; b) are shown for the immobilization (orange) and control (blue) groups before (Pre) and after (Post) the intervention period. Data are presented as median with interquartile range (box) and minimum‐to‐maximum values (whiskers). Individual data points represent each of the participants. Exact *p*‐values are shown for statistically significant within‐ and between‐group comparisons.

In contrast, analysis of relative force steadiness, quantified as the CoV%, revealed no main effect of Time (*F*(1, 26) = 0.03, *p* = 0.86) and no main effect of Group (*F*(1, 26) = 0.62, *p* = 0.44), but a significant Group × Time interaction (*F*(1, 26) = 8.04, *p* = 0.009; Cohen's *d* = 1.07). Post hoc comparisons indicated that the CoV% increased following immobilization (mean change: 0.21 CoV%, 95% CI [0.01, 0.41], *p* = 0.043; Figure 4a), whereas no significant change was observed in the control group (*p* = 0.071).

### 3.4 Effects of short‐term immobilization on manual dexterity

**FIGURE 5:**
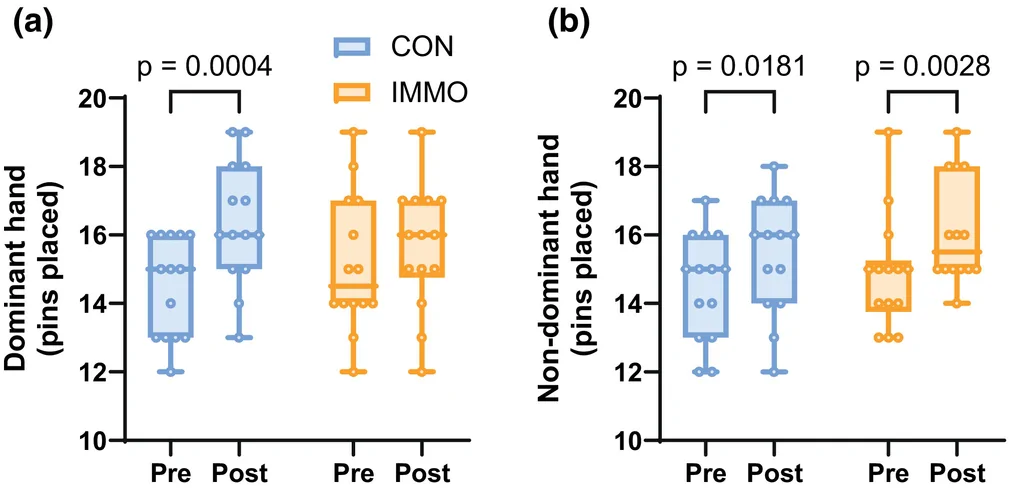
Effects of short‐term hand immobilization on manual dexterity. Changes in Purdue Pegboard Test performance for the dominant (a) and non‐dominant (b) hands are shown for the immobilization (orange) and control (blue) groups before (Pre) and after (Post) the intervention period. Data are presented as median with interquartile range (box) and minimum‐to‐maximum values (whiskers). Individual data points represent each of the participants. Exact *p*‐values are shown for statistically significant within‐ and between‐group comparisons.

For the dominant hand, analysis revealed a significant main effect of Time (*F*(1, 26) = 13.10, *p* = 0.0013), no main effect of Group (*F*(1, 26) = 0.004, *p* = 0.95), and a significant Group × Time interaction (*F*(1, 26) = 4.34, *p* = 0.047; Cohen's *d* = 0.79). Post hoc comparisons revealed a significant improvement from pre‐ to post‐test in the control group (mean change: 1.86 pins, 95% CI [0.91, 2.80], *p* = 0.0004; Figure 5a), whereas no significant change was observed in the immobilization group (*p* = 0.29).

For the non‐dominant hand, a significant main effect of Time was observed (*F*(1, 26) = 16.96, *p* = 0.0003), with no main effect of Group (*F*(1, 26) = 0.82, *p* = 0.37), and no Group × Time interaction (*F*(1, 26) = 0.30, *p* = 0.59). Post hoc analyses showed significant improvements from pre‐ to post‐test in both the control (mean change: 0.93 pins, 95% CI [0.17, 1.69], *p* = 0.018; Figure 5b) and immobilization group (mean change: 1.21 pins, 95% CI [0.46, 1.97], *p* = 0.0028).

## 4 DISCUSSION

The present study shows that even short periods of limb immobilization can induce domain‐specific impairments in motor performance. Specifically, immobilization led to marked reductions in both maximal and explosive strength, confirming that these aspects of motor function are particularly sensitive to short‐term immobilization. In contrast, no statistically significant changes were observed in the selected parameters of fatigability during sustained maximal contractions, suggesting that this aspect of motor performance may be less susceptible to short‐term immobilization. Immobilization selectively impaired relative but not absolute force variability, and manual dexterity was not significantly affected by immobilization. However, immobilization prevented the retest‐related performance improvements observed in both the control group and the contralateral non‐immobilized hand. Together, these findings highlight that only a few days of immobilization are sufficient to induce measurable deficits in specific motor domains, with maximal and explosive strength being particularly susceptible to adverse consequences.

### 4.1 Short‐term immobilization induces marked reductions in maximal and explosive strength

In line with previous studies, short‐term immobilization induced clear and robust reductions in both maximal and explosive strength (Campbell et al., 2019; Marusic et al., 2021; Ruggiero & Gruber, 2024). The magnitude of strength loss observed here is comparable to earlier reports of upper limb disuse following short periods of immobilization (Lundbye‐Jensen & Nielsen, 2008; Ngomo et al., 2012; Seki et al., 2007), confirming that even brief immobilization substantially compromises force‐generating capacity.

Explosive strength was similarly affected, with immobilization leading to significant reductions in RFD over both the early (0–50 ms) and later (0–200 ms) phases of force development. These findings suggest that the effects of short‐term immobilization extend across the force‐time curve rather than being confined to a specific phase of force generation. Consistent with previous studies, the mean percentage reduction in absolute RFD (0–50 ms: 26.1%; 0–200 ms: 26.4%) exceeded that observed for MVC (21.1%). However, the relative difference between the changes in RFD and MVC observed in the present study (approximately 5 percentage points) was smaller than that reported previously. For example, Monti et al. (2021) and Sarto et al. (2022) demonstrated substantially larger dissociations between changes in explosive and maximal strength following 10 days of bed rest and lower‐limb immobilization, with percentage‐point differences of 23.5 and 24.9, respectively, as summarized by Ruggiero and Gruber (2024). However, these studies quantified explosive strength using time‐to‐force threshold metrics, such as time to 63% of peak force, whereas the present study quantified explosive strength using the slope of the force‐time curve. Such methodological differences may account, at least in part, for the smaller dissociation observed in this study. Supporting this interpretation, Hvid et al. (2014) reported a difference of 5.6 percentage points following four days of lower‐limb immobilization using an RFD measure comparable to that employed here, closely matching the magnitude of the present findings. Although the percentage reduction in absolute RFD exceeded that observed for MVC, normalization of RFD to MVC revealed no statistically significant changes for either the 0–50 ms or 0–200 ms intervals, suggesting that the reduction in rapid force production was largely proportional to the concurrent decline in maximal force capacity. These findings indicate that short‐term immobilization substantially compromises the ability to generate force rapidly in absolute terms but do not provide evidence for a disproportionate impairment in explosive strength beyond that expected from the reduction in MVC.

Although the detrimental impact of immobilization on muscle strength is well established, surprisingly few studies have examined the effects of short‐term immobilization lasting less than 1 week, and the available evidence is largely derived from investigations of the lower limbs (Hvid et al., 2013, 2014; MacLennan et al., 2021). Accordingly, recent literature syntheses have compared changes in MVC and RFD almost exclusively in the lower limb muscles (Ruggiero & Gruber, 2024). To our knowledge, this is the first study to assess both MVC and RFD following short‐term upper extremity immobilization, demonstrating that patterns previously observed in the lower limbs also extend to the distal upper‐limb muscles. This is particularly relevant given that upper‐limb injuries are at least as prevalent as lower‐limb injuries and occur frequently in the hands (Sytema et al., 2010). Considering the functional importance of the human hand in daily activities, these findings emphasize the importance of protecting and restoring hand muscle strength in rehabilitation contexts.

### 4.2 No detectable changes in fatigability‐related parameters despite pronounced strength loss

The effects of immobilization on fatigability differed markedly from its pronounced impact on maximal and explosive muscle strength. In the present study, no detectable effects were observed on either the fatigue time constant or the level of force sustained relative to the initial capacity. Previous studies examining fatigability following immobilization have yielded mixed findings, partly due to substantial methodological heterogeneity. These studies have employed outcome measures such as time to task failure (Clark, Hoffman, & Russ, 2008) or rate of force decline (Duchateau & Hainaut, 1991), reporting decreased (Shaffer et al., 2000), unchanged (Fuglevand et al., 1995), or even improved fatigue resistance (Clark, Hoffman, & Russ, 2008; Semmler et al., 2000; Yue et al., 1997). The interpretation of these findings is further complicated by the fact that fatigability is commonly assessed during sustained contractions performed at a fixed percentage of the MVC force (Clark, Hoffman, & Russ, 2008; Semmler et al., 2000; Yue et al., 1997). Because MVC decreases following immobilization, the absolute force corresponding to a given relative intensity is substantially lower after a period of disuse. Although this normalization allows for the comparison of relative effort across sessions, it simultaneously reduces the absolute metabolic and mechanical load imposed on the muscle. Consequently, the reported improvements in fatigue resistance observed in some studies (Clark, Hoffman, & Russ, 2008; Yue et al., 1997) may reflect a reduced absolute workload rather than genuine alterations in fatigue‐related processes.

In contrast, the present study employed a sustained MVC task in which participants were instructed to maintain a maximal effort continuously for 2 min. Under these conditions, the absence of significant effects on fatigue‐related parameters suggests that short‐term immobilization did not induce marked changes in the mechanisms governing the progression of fatigue. Given that immobilization induces both neural and muscular maladaptive changes within days and markedly reduces maximal muscle strength (MacLennan et al., 2021; Mulder et al., 2015; Suetta et al., 2012; Wall et al., 2014), the absence of statistically significant changes in the selected fatigability parameters during sustained maximal contractions is somewhat surprising. However, this finding is consistent with previous behavioral evidence showing preserved fatigue trajectories following prolonged thumb immobilization, in which the rate of force decline during sustained MVC remained unchanged (Duchateau & Hainaut, 1991). Taken together, these findings suggest that short‐term immobilization has a greater impact on maximal and explosive strength than on the fatigability parameters assessed in the present study.

### 4.3 Immobilization impairs relative, but not absolute, force steadiness

In contrast to maximal contractions, submaximal force steadiness tasks assess the fine regulation of neural drive required to maintain accurate low‐amplitude force production and are essential for the execution of fundamental motor skills and everyday motor activities (Pethick et al., 2022). At the behavioral level, force steadiness reflects the fluctuations in force output over time and is closely linked to variability in the common synaptic input to the motor neuron pool (Farina & Negro, 2015; Feeney et al., 2018). As such, force steadiness provides a marker of motor control that is not captured by maximal force tests (Enoka & Farina, 2021).

Our findings showed that immobilization led to an increase in the CoV%, indicating reduced relative force steadiness. Expressing force variability as the CoV% accounts for changes in the mean force and therefore provides an index of steadiness relative to the target level. The observed increase in relative force variability following immobilization is consistent with previous reports of impaired steadiness after periods of disuse or unloading (Inns et al., 2022; Valli et al., 2024; Yoshitake et al., 2007). This suggests that immobilization may compromise the efficiency of force control mechanisms involved in steady force regulation.

Immobilization did not affect the absolute magnitude of force fluctuations, as quantified by the SD, indicating that the ability to sustain a submaximal target force in absolute terms was preserved. Accordingly, the impairment in steadiness appeared only when variability was expressed relative to the lower target force resulting from the reduction in the MVC. This dissociation between absolute and relative measures of force steadiness is consistent with observations in clinical populations with peripheral musculoskeletal conditions (Pethick et al., 2022) and older adults (Enoka et al., 2003), both of whom are characterized by reduced muscle strength compared with healthy or younger individuals, respectively. In such contexts, the CoV% is used to enable comparisons of force steadiness between conditions or groups that differ in strength (Pethick et al., 2022), such as following immobilization. Therefore, the selective increase in relative force variability is consistent with expectations when strength differs across conditions.

Some considerations should be taken into account when interpreting these findings, as the observed increase in relative force steadiness may not exclusively reflect impaired motor control. A key issue is that the CoV% is strongly influenced by absolute force level, with relative force variability increasing substantially at low force levels (Enoka & Farina, 2021; Galganski et al., 1993). Because immobilization reduced MVC, the post‐intervention target force corresponded to a lower absolute force, thereby shifting the contraction into a region of the force variability relationship where the CoV% is typically higher (Enoka & Farina, 2021). Accordingly, it cannot be excluded that the reduced target force contributed, at least in part, to the observed increase in CoV%. Therefore, the decrease in relative force steadiness may reflect a combination of motor impairment and an operating‐point shift along the force‐variability curve, rather than a pure change in force control processes.

Reduced force steadiness may have functional relevance beyond isometric tasks, as the ability to generate stable and precise submaximal forces is fundamental to coordinated movements. Measures of force steadiness have been suggested to be more closely related to performance in everyday motor activities than maximal muscle strength alone (Enoka & Farina, 2021; Pethick et al., 2022). Thus, although the reduction in force steadiness following immobilization was modest compared with the pronounced impairments in maximal and explosive strength, its functional consequences may nevertheless be meaningful.

### 4.4 Immobilization attenuates retest‐related improvements in manual dexterity

Manual dexterity was assessed using the Purdue Pegboard Test to capture aspects of motor function that extend beyond isometric force control. In the present study, short‐term immobilization did not result in a significant decline in pegboard performance of the dominant, immobilized hand, as performance remained stable from pre‐ to post‐test. This indicates that immobilization did not directly impair manual dexterity relative to baseline. Importantly, the inclusion of a non‐immobilized control group allowed for the evaluation of potential test–retest effects. In this group, significant improvements in dominant hand performance were observed, consistent with practice‐related gains. Similar improvements were also evident in the non‐dominant hand in both groups. These findings suggest that although immobilization did not reduce dexterous performance per se, it attenuated the retest‐related improvements observed under control conditions.

The improvement observed in the control group and hand is consistent with well‐documented practice effects in pegboard performance (Garry et al., 2004), reflecting motor learning and the optimization of movement strategies (Dayan & Cohen, 2011; Krakauer et al., 2019). The absence of comparable improvements following immobilization suggests that disuse interfered with the processes supporting performance gains across repeated tests. These findings align with those reported by Opie et al. (2016), who showed that short‐term hand immobilization did not impair baseline pegboard performance but disrupted the retention of previously acquired performance improvements. It has been proposed that immobilization may limit skill retention by altering neural plasticity processes involved in motor learning and memory, thereby constraining the consolidation of practice‐related improvements (Opie et al., 2016). Alternatively, it is possible that learning‐related changes still occurred in the immobilized system but were constrained by subtle impairments in motor control. These constraints may be related to the same control processes implicated in reduced force steadiness. Indeed, short‐term immobilization has been shown to alter inter‐joint coordination (Moisello et al., 2008), impair postural control of the arm and shoulder (Bolzoni et al., 2012), and disrupt motor planning (Scotto et al., 2026). Although these aspects were not directly assessed in the present study, it is possible that immobilization affected specific components of motor control that masked retest‐related improvements in dexterous performance. Nevertheless, the absence of a significant decline in pegboard performance suggests that manual dexterity was largely preserved following immobilization, particularly when contrasted with the pronounced reductions in maximal and explosive strength.

### 4.5 Immobilization is associated with domain‐specific motor impairment

Consistent with previous literature, immobilization was associated with negative consequences for performance across motor domains. However, existing evidence is largely derived from studies examining isolated aspects of motor control in separate participant samples, making it difficult to infer the relative susceptibility of different motor functions to disuse effects. While a multitask approach introduces potential challenges related to cross‐task influences and task order, the motor test battery employed in the present study allowed the assessment of how short‐term immobilization affects distinct motor functions within the same individuals.

**FIGURE 6:**
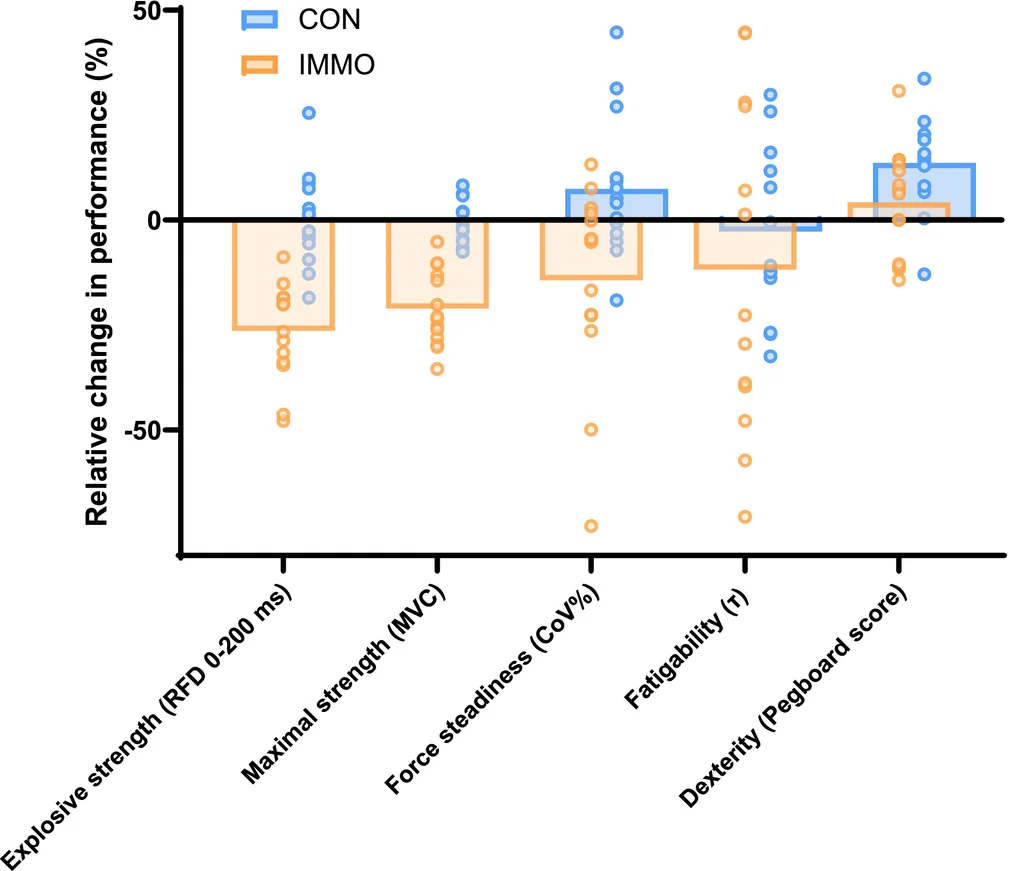
Relative changes in performance across motor domains following short‐term immobilization. Bars show mean percentage changes from baseline for the immobilization (orange) and control (blue) groups across motor domains, with individual participant values shown as open circles. For force steadiness, changes in coefficient of variation were sign‐inverted so that negative values consistently indicate reduced performance. Values are descriptive and provided for visual comparison.

The present findings demonstrate that short‐term immobilization induces task‐specific changes in motor performance, rather than a uniform impairment across domains (Figure 6). MVC and RFD showed the largest and most consistent impairments, indicating that maximal and explosive strength are particularly susceptible to the negative effects of short‐term disuse. Submaximal force steadiness also exhibited selective increases in relative force variability following immobilization. Although the reduction in force steadiness was smaller in magnitude than the decline in strength, the relative changes were more comparable when accounting for changes observed in the control group. While impairments in muscle strength and rapid force production are widely recognized as functionally relevant, force steadiness of the hand muscles is important for motor performance and may predict fine motor control, while force steadiness in the lower limbs has been linked to walking performance (Enoka & Farina, 2021), suggesting that immobilization‐induced changes in this domain could have functional relevance. However, evidence from immobilization studies suggests that deficits in force steadiness recover quickly and may even exceed baseline levels following reexposure to normal activity, whereas strength declines are not readily reversed and may require targeted activities to ensure fast recovery (Suetta et al., 2009; Valli et al., 2024). Moreover, effect size calculations revealed very large immobilization effects for maximal and explosive strength, with mean differences between groups exceeding two standard deviations, whereas moderate effect sizes were observed for relative force steadiness. This highlights the pronounced impact of immobilization on muscle strength compared to other motor domains. Similarly, moderate effect sizes were observed for manual dexterity. Notably, manual dexterity was not directly impaired by immobilization, but immobilization attenuated retest‐related performance improvements, resulting in negative effects on fine motor control. This effect is further emphasized by the observation that fine motor performance improved not only in the control group but also in the contralateral non‐immobilized hand. This effect may be related to impaired force steadiness, as increased force variability has been shown to predict manual control performance, including pegboard tasks (Marmon et al., 2011). In contrast, short‐term immobilization did not produce statistically significant changes in the fatigue parameters assessed during sustained maximal contractions, suggesting that the processes governing force maintenance over time may remain largely intact during disuse.

### 4.6 Limitations

Several limitations of the present study should be acknowledged. First, the study was designed as an exploratory multidomain assessment of motor function following short‐term immobilization and included multiple outcome measures representing different aspects of motor performance. Because these outcomes were analyzed separately and no single predefined primary outcome was specified, the findings should be interpreted with appropriate caution. In addition, although the sample size was comparable to previous immobilization studies assessing maximal and explosive strength (Campbell et al., 2019; Ruggiero & Gruber, 2024), larger sample sizes may have been preferable in other motor domains characterized by greater variability and smaller effect sizes. Furthermore, RFD was derived from the same contractions used to assess MVC and contraction onset was identified using a predefined automated threshold corresponding to 0.2% of peak force. Although these methodological choices were applied consistently across all participants and time points, alternative RFD protocols and onset detection procedures may yield different absolute RFD values and should be considered when comparing the present findings with those of other studies.

Second, the present study assessed only behavioral outcomes and did not include direct physiological measurements capable of identifying the mechanisms underlying the observed changes in motor performance. Although physiological adaptations following immobilization are ultimately expressed through alterations in motor behavior, the present findings cannot directly link specific physiological changes to their functional consequences.

Third, the 72 h immobilization period represents a relatively short‐term model of disuse and may not fully reflect the longer durations often encountered in some clinical settings. Nevertheless, measurable neuromuscular and functional changes are known to occur rapidly following short periods of immobilization (MacLennan et al., 2021), supporting the relevance of short‐term models for investigating early disuse‐related adaptations.

## 5 CONCLUSION

In conclusion, the findings highlight that short‐term immobilization preferentially affects motor domains associated with maximal and explosive force production. More moderate effects are observed in force steadiness and dexterous task execution, either through impaired performance or attenuated motor learning, whereas fatigability is relatively preserved. Accordingly, the present results suggest that, all else being equal, rehabilitation strategies should prioritize the protection and recovery of maximal and explosive strength, which appear especially vulnerable to short‐term disuse.

## AUTHOR CONTRIBUTIONS

Conceptualization: L.J., A.L.K., J.R.B., J.L.‐J.; Data curation: L.J.; Formal analysis: L.J.; Funding acquisition: L.J., J.L.‐J.; Investigation: L.J.; Methodology: L.J., A.L.K., J.R.B., J.L.‐J.; Project administration: L.J., J.L.‐J.; Supervision: J.L.‐J.; Validation: L.J.; Visualization: L.J., A.L.K., J.R.B., J.L.‐J.; Writing – original draft: L.J.; Writing – review and editing: L.J., A.L.K., J.R.B., J.L.‐J.

## FUNDING INFORMATION

L.J. and J.L.‐J. are supported by a grant from the Danish Ministry of Culture (SUAKPKfor2W.2022‐054). A.L.K. and J.L.‐J. are supported by a grant from Team Danmark through the Novo Nordisk Foundation under Grant Number (NNF.22SA0078293). J.R.B. and J.L.‐J. are supported by a grant from the Novo Nordisk Foundation (NNF22SA0081765). The funders had no role in the design, data collection and interpretation, or decision to submit the manuscript for publication.

## CONFLICT OF INTEREST STATEMENT

The authors declare no conflicts of interest.

## Supporting information


**Table S1.** Questionnaire used during fieldwork including dataset variable description.

## Data Availability

The data of this study are available upon request by contacting the corresponding author.
