# Supplementary material for: Effects of short‐term hand immobilization on motor functions across domains in adults
Source: Physiol Rep. 2026 Aug 20;14(16):e71068. doi: 10.14814/phy2.71068 (PMC13494355; doi:10.14814/phy2.71068)
Supplement: Supplementary file 1 [file PHY2-14-e71068-s001.pdf]

TABLE A1 Questionnaire used during fieldwork including dataset variable description.

PARAMETER	SURVEY QUESTION
Province, District, Sector, Cell, & Village	Corresponds to administration levels 1–5, respectively.
Antierosio	Is there any antierosion activity on this plot?
Antieros_1	Types of anti-erosion activities existing on this plot.
Other1	Any other type of anti-erosion activity?
Agroforest	Is there any agroforestry practise on this plot?
Agro_type	Types of agroforestry trees planted in this plot.
Other2	Any other type of agroforestry trees planted?
Cur_season	What is the current season?
Crop_sys	What is the cropping system?
landcover	Final crop type label amongst the crops of interest (bean,irish potato,maize, or rice).
status	Status of the point after quality control, indicating whether it was used in the creation of a plot polygon (used) or excluded (not used).
Crop_plan	What crop is planted currently/observed crop/
Mixed_crop	If crop observed is mixed, list here the mixed crops.
Crop_area	What is the area covered by the crop currently planted/observed crop (in are)?
Plant_date	What was the planting date of the crop?
Growth_stg	At what growth stage is the crop?
Har_season	Will this crop be harvested in this agricultural season?
Har_period	What is the expected period for harvesting this crop?
All_crops	Enter all current season crops around the farm (maximum 5)?
SA_crop	Which crop did you plant for Season A (NB: Where current observed crop is)?
SA_prod	What was your production for Season A (in kg per are)?
SB_yield	What is your expected yield for Season B (per are) for crop currently planted/observed crop?
Var_reason	What is the reason for variation in yields?
Land_conso	Is this plot located in land consolidated site during this agricultural season?
Crop_propo	What is the crop proportion (relative abundance) of the main crop?
Seed_var	Which seed variety has been planted?
Fert_type	Which type of fertilizer do you use?
Base_fert	What is the name of base fertilizer used?
Base_quant	How much of this fertilizer did you apply (kg per are)?
Topdr_fert	What is the name of topdress fertilizer used?
Topdr_quant	How much of this fertilizer did you apply (kg per are)?
Fruit	Is there any type of fruit in this plot (only scattered fruit tree)?
Fruit_typ	What kind of fruits (fruit name) are in this plot?
fruitname_	Any other type of fruit trees planted?
Fruit_numb	How many fruits trees are in this plot for each type of fruit trees?
Farm_reasn	Do you farm for subsistence or commercial or both?
Weeding	How do you carry out weeding?
Water_sour	What is your main watersource for farming?
Harvest	How do you harvest your crops?
GPS_Lat and GPS_Long	GPS latitude and longitude coordinates value (automatically captured).
GPS_Elev	GPS elevation value in meters.
GPS_Prec	GPS precision value in meters.
North_pho , East_pho, West_pho, & South_pho	Photos facing North, East, West, & South directions, respectively.
Comment	Additional Comment
F_id	Default KoboToolbox identification value.
F_uuid	Unique identifier value.
F_submissi	Submission date.
F_version	Default KoboToolbox version.
F_index	Default.
Field	Default.
